# Supplementary material for: Efficient computation of adjoint sensitivities at steady-state in ODE models of biochemical reaction networks
Source: PLoS Comput Biol. 2023 Jan 3;19(1):e1010783. doi: 10.1371/journal.pcbi.1010783 (PMC9838866; doi:10.1371/journal.pcbi.1010783)
Supplement: Attachment — Submitted filename: response_to_reviewers.pdf [file pcbi.1010783.s002.pdf]

Dear Dr Attila Csikász-Nagy,
Dear Dr Mark Alber,
Dear anonymous reviewers,
We thank you for your constructive comments on our manuscript *“Efficient computation of adjoint sensitivities at steady-state in ODE models of biochemical reaction networks”*. Based on your remarks, we have revised the manuscript. We addressed all comments and tried to resolve the raised issues. Our point-by-point response is provided below.

The reviewers’ comments are highlighted by italics, while the changes made in the manuscript are highlighted by yellow.

Response to Reviewer 1

The paper by Lakriscnko et al presents a new numerical algorithm for sensitivity analysis of ODE models, mostly applied to biochemical reaction networks. They focus on in particular speeding up situations where steady-state data are available and of interest, in addition to dynamic data as typically. Overall the manuscript is well written, easy to follow, and presents a significant advance in this class of problems, particularly for larger models where the differences of speed are important, and which are becoming more and more prevalent. I only have some minor comments that the authors may consider in a revision.

We thank Reviewer 1 for their positive feedback.

1. *First, I’d like to have seen a little more analysis or speculation / discussion as to why speed up is bigger for some models and not others, and how this speed up scales with problem sizes. Fig. 4 and 6 show some of this data but one is left wondering if there is any insight for future models, what the speed up may be based on certain properties.*

   Response:
   The speedup depends on the computational complexity of computing the integral (12) (or (16) for pre-equilibration case) on the time interval between the last finite time point and the time point where the steady-state condition is satisfied. It is the difference between solving an n_x-dimensional linear non-autonomous ODE system compared to solving a linear system of n_x equations. The speedup is expected to be higher for nonlinear systems. It doesn’t depend on the number of optimized parameters as shown in Fig. 5.

   It also depends on what fraction of the available data are steady-state measurements. If mostly time-course data is available the speedup will not be as significant as when predominantly steady-state data is used.

   The speedup also depends on what fraction of overall computational complexity comprises the integral computation, as discussed at the end of the Results Section.

   We have now added these explanations to the Discussion section of the manuscript.

Changes in the manuscript:
Initial submission:
The exact speedup over the standard approach depends on the model and dataset. We observed a speedup of up to 3.3-fold for simulations and up to two-fold for parameter estimation, for the models and datasets considered in this study.

Revised submission:
The exact speedup over the standard approach depends on the model and dataset. More specifically, it depends on the computational complexity of computing the integral (\ref{eq:adjoints_integral}) (or (\ref{eq:preeq_quadrature}) for pre-equilibration case) on the time interval between the last finite time point (t_{n_t}) and the time point where the steady-state condition is satisfied. It is the difference between solving an n_x-dimensional linear non-autonomous ODE system compared to solving a linear system of n_x equations. The speedup is expected to be higher for nonlinear systems. It also depends on the fraction of computational cost spent on the steady-state. For example, if the time spent in the optimizer is large compared to the time for model simulation, or if mostly time-course data is available, the speedup will be lower. As expected, the speedup does not depend on the number of optimized parameters, which we demonstrated with the example of one of the three models. We observed a speedup of up to 3.3-fold for simulations and up to two-fold for parameter estimation, for the models and datasets considered in this study.

2. *A key weakness was inapplicability to models with conserved quantities (singular Jacobian). It would have also been nice to see the implementation on a model that had a singular Jacobian due to conserved quantities, but the tools being mentioned as solutions to this caveat (e.g. Ref 35) were applied and then the presented approach was successfully employed.*

   Response:
   As the Reviewer pointed out correctly, the proposed method cannot be applied to models with conserved quantities. This was the case for one of the three considered models, namely Blasi et al., 2016 model, and, therefore, applying the introduced ssASA approach directly is not possible. Therefore, we have implemented the heuristic-based conserved moieties identification approach (Ref. [35]) in the AMICI package.
   For the Blasi et al., 2016 model, conserved quantities were removed during model import by AMICI to ensure non-singularity of the Jacobian. Running our implementation on the unprocessed model would simply raise an error and we would not be able to show any results.

   We have further highlighted this in the Discussion and Conclusion section.
   Changes in the manuscript:
   Initial submission:
   In order to facilitate applicability of the proposed method we have implemented a fast heuristic-based approach for conserved quantities identification suitable for large-scale reaction networks as presented in \cite{DeMartino2014}. Both the

conserved moieties identification approach and the proposed ssASA approach have been made available via the open-source AMICI package.

Revised submission:
In order to facilitate applicability of the proposed method we have implemented a fast heuristic-based approach for conserved quantities identification suitable for large-scale reaction networks as presented in \cite{DeMartino2014}. Both the conserved moieties identification approach and the proposed ssASA approach have been made available via the open-source AMICI package.
We have tested this case on the Blasi et al., 2016 model. Conserved quantities have been removed before applying the proposed ssASA approach.

Response to Reviewer 2

This paper proposes a useful approach for the sensitivity computation of dynamical models taking into consideration steady state data. It is an appealing property of the method that the computation burden does not depend on the number of parameters. The advantages of the approach over existing popular methods are well demonstrated on the case studies taken from the literature. The supporting information is also useful for better understanding. My main overall comment is that the assumptions, notations and applicability conditions should be made more precise in the paper before publication.

Specific comments:

1. *The title contains 'biochemical reaction networks' although the proposed method does not seem to be specific to reaction networks or even to nonnegative biological models. Please clarify the system class to which the methodology is applicable.*

   Response:
   This is indeed the case, the proposed method is not tailored to biological applications, and, therefore, also useful beyond the field of computational biology. Taking into account this comment, we have highlighted this in the “Methods” and “Discussion and Conclusion” sections.

   Changes in the manuscript:
   Methods, initial submission, line 73:
   In this section, we introduce the considered classes of mathematical models and parameter estimation problems. Subsequently, we describe the established method for gradient calculation via adjoint sensitivity analysis (ASA) and propose a new formulation for the steady-state case.

   Methods, revised submission, line 73:
   In this section, we introduce the considered classes of mathematical models and parameter estimation problems. Subsequently, we describe the established method for gradient calculation via adjoint sensitivity analysis (ASA) and propose a new formulation for the steady-state case. We discuss how the method can be applied to models of biochemical reaction networks. The method, however, is not restricted to

the field of computational biology. It can be used for any model of the form described in this section with pre- or post-equilibration, or both.

Methods, initial submission, line 81:
We consider ODE models of biochemical processes of the form

Methods, revised submission, line 81:
We consider ODE models of the form

Methods, initial submission, line 85:
The ODE model determines the time evolution of the state variables, e.g., concentrations of biochemical species, given the parameters, e.g., reaction rate constant.

Methods, revised submission, line 85:
The ODE model determines the time evolution of the state variables given the parameters. In case of biochemical reaction networks, this can be e.g., concentrations of biochemical species and reaction rate constants, respectively.

Discussion and Conclusion, initial submission:
We tested the new approach on three published models and demonstrated that it is accurate and allows for efficient sensitivity computation.

Discussion and Conclusion, revised submission:
We described how the new approach can be applied in the case of modelling biochemical processes, tested it on three published models and demonstrated that it is accurate and allows for efficient sensitivity computation.

2.

2.1 The notation in eq. (1) is odd: if we want to denote all arguments correctly, I think $\dot{x} = f(x(t), \theta, u(t))$ *would be sufficient provided that the input is time-varying and the parameters are assumed to be constant. This makes* x *to depend on* θ *and* u *since the derivative depends on them.*

Response:
We corrected the notation as suggested.

Moreover:
2.2
- It is not clear whether the input is constant or time-varying. Can it be considered as a vector of known parameters?
Response:
We assume that input is constant and can be considered as a vector of known parameters. We have clarified that in the Mathematical modeling and simulation subsection.

Changes in the manuscript:
Initial submission:
… in which $\mathbf{x}(t, \bm{\uptheta}, \mathbf{u}) \in \mathbb{R}_+^{n_x}$ is the vector of state variables, $\bm{\uptheta} \in \mathbb{R}_+^{n_\theta}$ is the parameter vector, $\mathbf{u}\in \mathbb{R}_+^{n_u}$ is the vector of inputs and $t \in \mathbb{R}$ is the time.

Revised submission:
… in which $\mathbf{x}(t, \bm{\uptheta}, \mathbf{u}) \in \mathbb{R}_+^{n_x}$ is the vector of state variables, $\bm{\uptheta} \in \mathbb{R}_+^{n_\theta}$ is the parameter vector, $\mathbf{u}\in \mathbb{R}_+^{n_u}$ is the vector of constant inputs and $t \in \mathbb{R}$ is the time.

2.3
- Why does the initial condition directly depend on the parameters? This is also unusual.

Response:
For example, it can be that initial concentrations of some biochemical species are estimated from the data, i.e. x_i(t_0) is considered as a parameter and is included in the vector \theta. For example, in the model by Bruno et al., which describes the activity of Arabidopsis carotenoid cleavage dioxygenase 4 activity, initial concentrations are estimated in addition to rate constants (https://doi.org/10.1093/jxb/erw356). Initial insulin receptor concentrations were estimated in the model for cellular insulin binding, internalization and extraction processes by Schwen et al. (https://doi.org/10.1371/journal.pone.0133653).

3. *Are there any assumptions on the allowed state and parameter dependence of the dynamical model? (e.g., (quasi)polynomial, rational etc.)*

Response:
There are no special assumptions on the allowed state and parameter dependence of the dynamical model, except Lipschitz-continuity of the right hand side.
We have added this assumption to the “Mathematical modeling and simulation” subsection.
Changes in the manuscript:
Initial submission:
The vector field of the ODE model is $\mathbf{f}: \mathbb{R}_+^{n_x} \times \mathbb{R}_+^{n_\theta} \times \mathbb{R}_+^{n_u}\rightarrow \mathbb{R}_+^{n_x}$ and the initial condition at t_0 is $\mathbf{x}_0: \mathbb{R}_+^{n_\theta} \times \mathbb{R}_+^{n_u} \rightarrow \mathbb{R}_+^{n_x}$.
Revised submission:
The vector field of the ODE model is $\mathbf{f}: \mathbb{R}_+^{n_x} \times \mathbb{R}_+^{n_\theta} \times \mathbb{R}_+^{n_u}\rightarrow \mathbb{R}_+^{n_x}$, which is Lipschitz-continuous with respect to x, and the initial condition at t_0 is $\mathbf{x}_0: \mathbb{R}_+^{n_\theta} \times \mathbb{R}_+^{n_u} \rightarrow \mathbb{R}_+^{n_x}$.

4. *Please comment on the applicability of the approach for models with steady state multiplicity, since these are quite common among biological systems.*

   Response:
   We thank the reviewer for this question. Indeed, there might be multiple steady states. Due to Lipschitz-continuity of the right hand side of the system, there is a unique mapping from initial conditions to steady state.
   Newton's method does not necessarily obey this mapping but might (in principle) converge to a wrong steady state (not the one that would be reached by starting a simulation at $\mathbf{x}_0$). In practice, we do not observe this, most likely because Newton's method yields a similar path as ODE solver. The appearance of a problem can easily be checked after optimization.
   If a problem occurs, a short simulation-based pre-equilibration can be performed to provide Newton's method with a starting point closer to the corresponding steady state.

   We have added the clarification to the Discussion and Conclusion section.

   Changes in the manuscript:
   Initial submission:
   The new approach is only applicable if the (transposed) Jacobian is non-singular. Otherwise, the adjoint state ODEs have to be integrated numerically. One of the main reasons for a singular Jacobian is the presence of conserved quantities in the dynamical system \cite{Oellerich2021}. In order to facilitate applicability of the proposed method we have implemented a fast heuristic-based approach for conserved quantities identification suitable for large-scale reaction networks as presented in \cite{DeMartino2014}. Both the conserved moieties identification approach and the proposed ssASA approach have been made available via the open-source AMICI package.

   Revised submission:
   The new approach is only applicable if for each initial value there exists a unique, exponentially stable steady state. This implies that the (transposed) Jacobian of the right-hand side is non-singular. One of the main reasons for a singular Jacobian is the presence of conserved quantities in the dynamical system \cite{Oellerich2021}. In order to facilitate applicability of the proposed method we have implemented a fast heuristic-based approach for conserved quantities identification suitable for large-scale reaction networks as presented in \cite{DeMartino2014}. Both the conserved moieties identification approach and the proposed ssASA approach have been made available via the open-source AMICI package. If the uniqueness of the steady state cannot be ensured, e.g. because the system might be multi-stable, the method has to be applied carefully. While there also is a unique mapping from initial condition to steady state for multi-stable systems with Lipschitz-continuous right-hand sides, this mapping might not be obeyed by Newton's method. We do not observe this problem in practice, but recommend to check this after optimization and to provide Newton's method with a refined initial value (e.g. obtained via a short simulation run, which is already supported by AMICI) if necessary.

5. *In Fig 1.a, the equilibrium x^* depends on u. This is suspicious. (Why not on u^e?)*

   Response:
   x_0(u) = x^* indicates that the initial condition for the system under the input u is set to be equal to a steady state of the same system under the input u^e. The value of x^*depends on u^e.
   Changes in the manuscript:
   Initial submission:
   Top: The pre-equilibration case, where the system is at steady state at the beginning of the experiment. This means that by $t=t_0$ the system has reached its steady state ($\mathbf{x}_0$) under some condition ($\mathbf{u}^e$) (blue line). At $t=t_0$ the system was perturbed and measurements were taken at time points $t_j > t_0$ (orange crosses). The pre-equilibration steady state ($\mathbf{x}_0$) is the initial state under the experimental conditions ($\mathbf{u}$).
   Revised submission:
   Top: The pre-equilibration case, where the system is at steady state at the beginning of the experiment. This means that by $t=t_0$ the system has reached its steady state ($\mathbf{x}^*$) under some condition ($\mathbf{u}^e$) (blue line). At $t=t_0$ the system was perturbed and measurements were taken at time points $t_j > t_0$ (orange crosses). The pre-equilibration steady state ($\mathbf{x}^*$) is the initial state under the experimental conditions ($\mathbf{u}$), i.e. $\mathbf{x}_0(\mathbf{u}) = \mathbf{x}^*$.
6. *On page 5 the authors write:*
   "The results also apply to unknown noise variances and other noise distributions."
   I think that at least the mean of the noise should be zero and the noise components should be uncorrelated at different time instants, otherwise the parameter estimation will be biased.

   Response:
   We agree with the reviewer and we clarified that at the end of the Experimental data subsection.
   Changes in the manuscript:
   Initial submission:
   Remark: For the subsequent sections we assume, without loss of generality, that the noise is normally distributed and the variance is known. The results also apply to unknown noise variances and other noise distributions.
   Revised submission:
   Remark: For the subsequent sections we assume, without loss of generality, that the noise is normally distributed and the variance is known. The results also apply to unknown noise variances and other noise distributions, given that measurements are independent and the mean of the noise is equal to zero.

Response to Reviewer 3

It is well known that inhomogeneous linear ODEs can be solved in two ways: (1) solving the equations directly; (2) solving the corresponding homogeneous linear ODE. The solution is called the adjoint function or Green's function. Then, the Green function is integrated with the inhomogeneous part, providing the solution of the original ODE.

George Green (14 July 1793–31 May 1841) was a British mathematician and physicist, who wrote "An Essay on the Applications of Mathematical Analysis to the Theories of Electricity and Magnetism" (Green, 1828). The essay introduced several important concepts, among them a theorem similar to modern Green's theorem, the idea of potential functions as currently used in physics, and the concept of what are now called Green's functions. *https://www.nottingham.ac.uk/physics/about/history/george-green.aspx*

1. *Green published this idea in 1828, therefore the following sentence of the authors is not well founded:*
   "This approach has been developed in [Plessix RE, Geophysical Journal International. 2006]. The first application of the adjoint function method (also called Green's Function Method, GFM) in chemical kinetics for the calculation of sensitivity coefficients were published by J. Hwang et al. (The Green's function method of sensitivity analysis in chemical kinetics, The Journal of Chemical Physics 69 (1978) 5180-5191.)
   I recommend the authors a recent paper about the calculation of sensitivity coefficients in chemical kinetics using the GFM: M. Lemke et al. Combustion Theory and Modelling, 23, 180-196 (2019), https://doi.org/10.1080/13647830.2018.1495845). In this paper, references [16]-[27] discuss the history of the GFM in the analysis of chemical kinetic models.

   Response:
   We agree with Reviewer 3 that the sentence is misleading and that at least for certain classes of systems and observations it has been introduced much earlier. We reformulated the sentence accordingly:

   Changes in the manuscript:
   Initial submission:
   For large-scale ODE models, the objective function gradient for time-course data $\mathcal{D}_t$ is usually calculated using ASA. This approach has been developed in \cite{Plessix2006} and later adopted to parameter estimation problems in biological applications in \cite{FroehlichKal2017} for the case of time-course measurements without pre- or post-equilibration. For a comprehensive description of ASA, readers are referred to \cite{FroehlichKal2017}.

   Revised submission:
   For large-scale ODE models, the objective function gradient for time-course data $\mathcal{D}_t$ is usually calculated using ASA. This approach has a long history and relates to Green's function method of sensitivity analysis (see e.g. \cite{M. Lemke et al. Combustion Theory and Modelling, 23, 180-196 (2019), https://doi.org/10.1080/13647830.2018.1495845} and references therein) and is used

across scientific disciplines. \citep{FroehlichKal2017} adopted it for parameter estimation in biological applications for the case of time-course measurements without pre- or post-equilibration. For a description of ASA, readers are referred to \cite{FroehlichKal2017}.

2. *The authors do not solve the differential equations, but calculate the sensitivity coefficients in the stationary point, when the ODEs are reduced to linear algebraic equations. These matrix equations were derived from the GFM concept. This is also an obvious step. The paper does not contain any new math and very little novelty from a computational point of view. However, I still consider the publication of the paper possible, since the authors well justify that the software they created and made publicly available can be useful for the interpretation and optimization of some systems biology measurements.*

   Response:
   We thank the reviewer for considering the software a valuable contribution. Concerning the methodological contribution, it is not clear to us how the calculation of the sensitivity coefficients for the stationary point can be viewed as being separated from solving a differential equation. As the stationary point is the initial condition for a simulation (with a potentially different vector field), both components are coupled in forward and backward equations. To clarify this, we added an explanation in the revised version of the manuscript.

   Changes in the manuscript:
   Initial submission:
   "For this, the adjoint state from the time-course adjoint simulation must be passed on to the initial adjoint state of backward pre-equilibration ($\mathbf{p}(t_0, \bm{\uptheta}, \mathbf{u})$)."

   Revised submission:
   "For this, the adjoint state from the time-course adjoint simulation must be passed on to the initial adjoint state of backward pre-equilibration ($\mathbf{p}(t_0, \bm{\uptheta}, \mathbf{u})$). Accordingly, for the case of pre-equilibration, the ODEs solved for pre-equilibration and time-dependent observations are coupled, and cannot be computed independently."

3. *Surely, selected papers from references [16]-[27] have to be added to the manuscript.*

   Response:
   We thank the reviewer for pointing us to this valuable literature.
   As we did not want to discuss the history of the method, we decided to rather refer to the work of Lemke et al. (Combustion Theory and Modelling, 23, 180-196 (2019), https://doi.org/10.1080/13647830.2018.1495845}) and references therein. We think that this can provide a more comprehensive picture than we ever could.

4. *"The new approach is only applicable if the (transposed) Jacobian is non-singular. Otherwise, the adjoint state ODEs have to be integrated numerically."*

I guess most real life models contain conserved quantities, making the Jacobian singular Jacobian is non-singular. I am not conviced that a speedup of 4.4 justifies all these efforts.

Response:
We agree with Reviewer 3 that conserved quantities occur commonly. As presented in the manuscript, there exist various algorithms to detect those conserved quantities. In our implementation, conserved quantities are automatically replaced in a preprocessing step, so that the proposed approach can be applied after all. Although a 4-fold speed-up might not seem huge, in practice it makes a significant difference whether parameter estimation takes 1 week or 4 weeks. For users of AMICI, the effort to use this approach is only changing 2 options, which seems more than reasonable for a 4-fold speed-up. This stresses also the importance of the implementation of the approach in an established framework.
